# GABAergic anxiolytic drug in water increases migration behaviour in salmon

**DOI:** 10.1038/ncomms13460

**Published:** 2016-12-06

**Authors:** Gustav Hellström, Jonatan Klaminder, Fia Finn, Lo Persson, Anders Alanärä, Micael Jonsson, Jerker Fick, Tomas Brodin

**Affiliations:** 1 Department of Ecology and Environmental Science, Umeå University, SE-901 87 Umeå, Sweden; 2 Department of Wildlife, Fish and Environmental Studies, Swedish University of Agricultural Sciences, SE-901 83 Umeå, Sweden; 3 Department of Chemistry, Umeå University, SE-901 87 Umeå, Sweden

## Abstract

Migration is an important life-history event in a wide range of taxa, yet many migrations are influenced by anthropogenic change. Although migration dynamics are extensively studied, the potential effects of environmental contaminants on migratory physiology are poorly understood. In this study we show that an anxiolytic drug in water can promote downward migratory behaviour of Atlantic salmon (*Salmo salar*) in both laboratory setting and in a natural river tributary. Exposing salmon smolt to a dilute concentration of a GABA_A_ receptor agonist (oxazepam) increased migration intensity compared with untreated smolt. These results implicate that salmon migration may be affected by human-induced changes in water chemical properties, such as acidification and pharmaceutical residues in wastewater effluent, via alterations in the GABA_A_ receptor function.

Animal migration, be it over land, in the oceans, or through the air, is an astonishing phenomenon that expose animals to extraordinary challenges as they travel into and across unknown and often risky environments. Although the ecological factors underlying migration have been extensively studied in many animals, the neurobiological mechanisms governing migration are still poorly understood. Atlantic salmon (*Salmo salar*) is an iconographic species with immense biological, cultural and socio-economic value^1^,^2^. Its life cycle includes a vast and critical migration event, starting with the juveniles (smolts) leaving their natal river, heading thousands of kilometres out into the ocean to feed and grow, returning a few years later to the same river to spawn as large adults^3^. Understanding what regulates salmon migration dynamics is imperative for effective management and conservation of salmon populations, especially in the face of declining populations^4^,^5^. One of the most critical phases of the migration cycle is when the smolts leave their nursery habitat in the river and migrate downstream to the ocean^6^. Although decades of research have identified many of the underlying environmental and ontogenetic factors influencing the onset and intensity of salmon smolt migration^4^,^7^,^8^, a deeper understanding of general mechanisms controlling migration is still lacking^4^,^9^. So far, no studies have looked beyond hard-wired environmental cause–effect relationships in their effort to explain migration behaviour in salmon.

Downstream migration is characterized by high risk and uncertainty for the smolt, as they enter an unknown and potentially dangerous environment, often full of predators^6^,^10^. In other migrating fish, risk-prone individuals have been found more willing to migrate than risk-averse individuals^11^, but little is known about the mechanistic process underlying such differentiation. Recently, attention to cognitive and psychological dimensions in how animals respond to risk and danger has provided intriguing new insights in behavioural ecology and management^12^,^13^. Sustained stress grounded in anticipation of fear and danger may permeate an individual's motivation, choices and behaviour, and has been found to influence a wide range of ecological characteristics, including species distribution and population demographics^13^, complementing more established ecological drivers such as resource availability and environmental conditions. Such anxiety-related emotion-based stress is thought to be adaptive, as it may minimize unnecessary risk taking and maintain alertness even though no immediate stressor, such as a predator, might be present^12^.

In vertebrates, anxiety can be reduced by activation of the ligand-gated GABA_A_ receptor in the central nervous system, either by the endogenous inhibitory neurotransmitter gamma-aminobutyric acid (GABA) or by other agonistic molecules, reducing overall neurotransmission by decreasing the neurons action potential^14^. The GABA_A_ receptor system is highly conserved within vertebrates^15^, and several psychiatric drugs intended to reduce anxiety in humans also have anxiolytic-like effects on other animals, such as rodents and fish^16^,^17^. In fish, where the relevance of cognitive and emotional concepts are intensely debated^18^,^19^, drugs targeting the GABA_A_ receptor function constitute powerful neurobiological tools to understand and interpret cognitive aspects of behavioural responses to risk and danger. Further, altered behaviour in fish due to modulation of the GABA_A_ receptor function following exposure to anthropogenic water pollution, such as pharmaceutical residues and/or acidification, has been highlighted as a major concern in aquatic ecotoxicology and conservation^17^,^20^.

In this study, we investigated whether exposure to environmental relevant concentrations of oxazepam, a positive allosteric modulator of the GABA_A_ receptor and a common agent in anxiolytic pharmaceuticals, affected the migration behaviour of Atlantic salmon smolt. Migration of smolts exposed to oxazepam in water was quantified and compared with non-exposed smolt migration both in the laboratory and in the field, by tracking the downstream movement of each individual smolt using small implanted microchips. In the laboratory, the intensity (that is, speed) of the migration was quantified as the number of downstream laps a smolt migrated in a large migration mesocosm. In the field, the migration speed was measured as the time it took for a smolt to migrate out of a small river tributary.

## Results

### Downstream migration intensity

**Figure 1:**
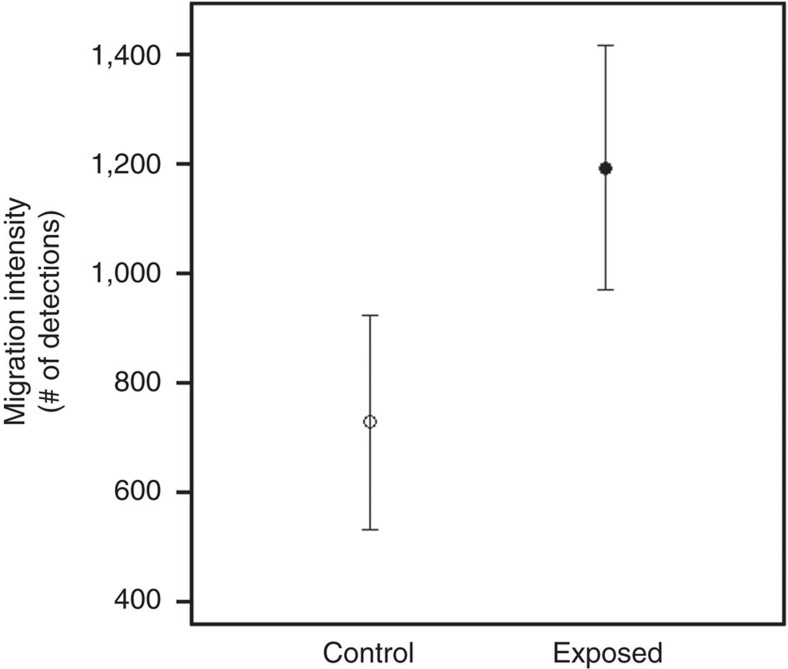
Effects of oxazepam on downstream migration in Atlantic salmon smolt. Downstream migration intensity for Atlantic salmon smolt (mean and 95% confidence intervals) in control and oxazepam-exposed Atlantic salmon smolts in a large-scale migration mesocosm. Migration intensity is inferred from the number of laps registered in the migration pool, and values for each treatment (exposed and control) represents the mean number of laps per smolt individual completed in the migration pool during 70 h, and is based on five independent replicated trials, each containing 20 smolts per treatment.

**Figure 2:**
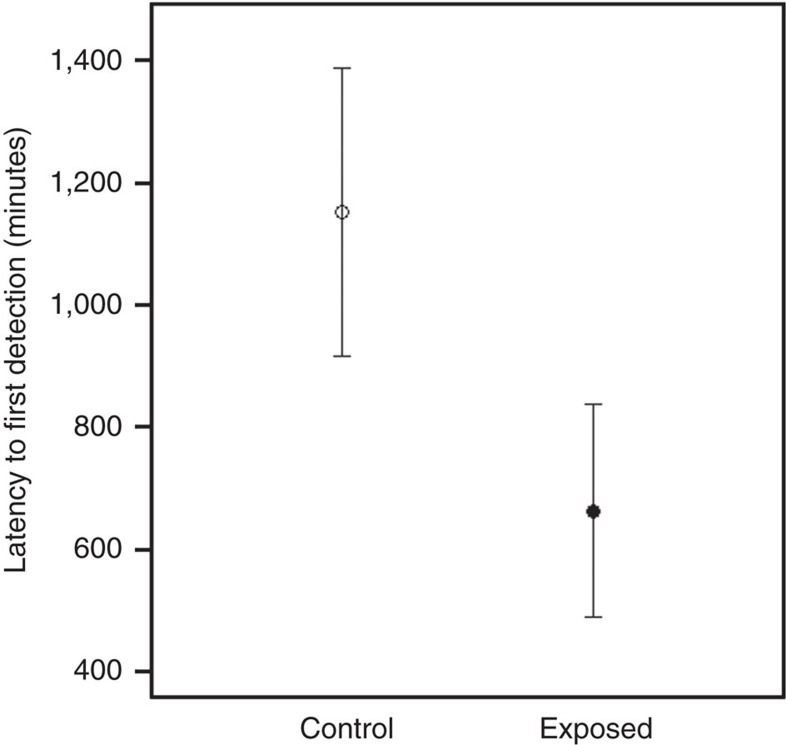
Effects of oxazepam on downstream migration in Atlantic salmon smolt. Downstream migration intensity of control and oxazepam-exposed Atlantic salmon smolt in a natural river tributary. Migration intensity was measured as latency to first detection (mean and 95% confidence interval) at the PIT-tag antenna 200 m downstream of the release site.

**Figure 3:**
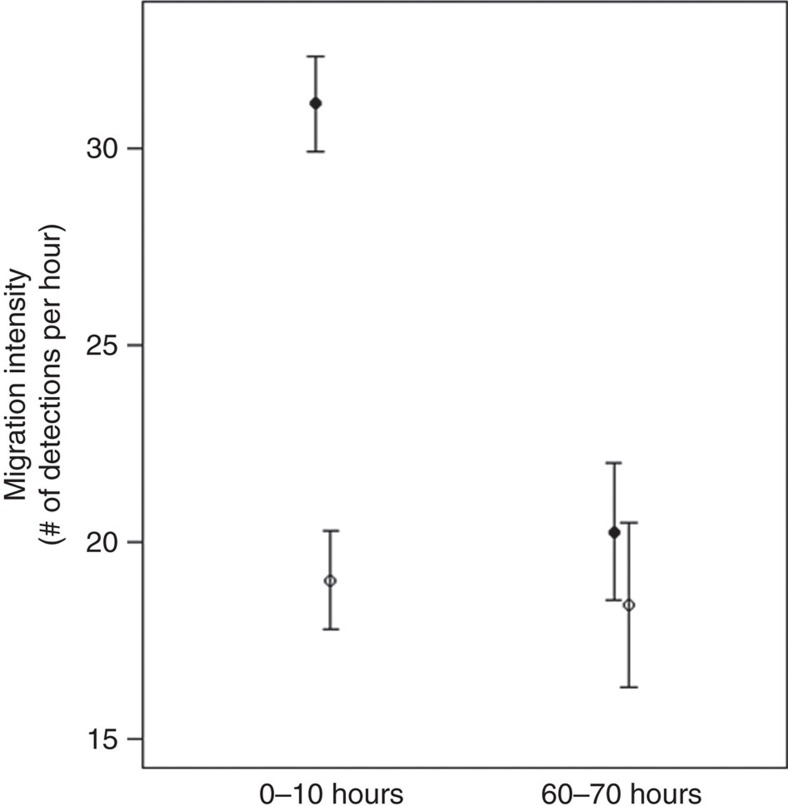
Downstream migration intensity in Atlantic salmon smolt 0–10 and 60–70 h after exposure to oxazepam. Downstream migration intensity (mean and 95% confidence intervals) in control (white) and oxazepam-exposed (black) Atlantic salmon smolts during the first 10 h of migration and the last 10 h (between 60 and 70 h) of migration in a large-scale migration mesocosm.

**Figure 4:**
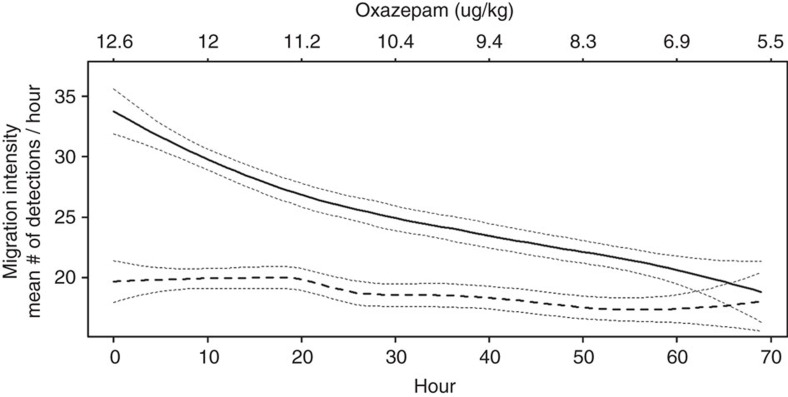
Downstream migration intensity in Atlantic salmon smolt over time and with decreasing concentration of oxazepam in fish muscle tissue: Downstream migration intensity in control (dashed line) and oxazepam-exposed (solid line) Atlantic salmon smolt as a function of time (lower *x* axis) and concentration of oxazepam in fish muscle tissue (upper *x* axis). Values are fitted values from a Loess smoother, and dotted lines are 95% confidence interval bands.

In both the laboratory and field study, smolts exposed to oxazepam migrated significantly faster than unexposed control fish (laboratory study: general linear mixed model (LME), F_1,170_=19.7, *P*<0.01, Fig. 1; field study: LME, F_1,107_=10.7, *P*=0.014, Fig. 2). The effect of treatment on migration intensity in the lab was dependent of time (that is, significant interaction term; LME, F_1,2259_=39, *P*<0.01), where migration intensity of exposed fish was significantly higher than of control fish during the first 10 h (*post hoc* Tukey, *P*<0.01, Fig. 3), but not during the last 10 h (that is, between 60 and 70 h; *post hoc* Tukey, *P*=0.53, Fig. 3) of the migration trials. Analysis of within-treatment changes in migration intensity over time showed no difference in migration intensity of control fish between the first 10 and the last 10 h of the migration trial (*post hoc* Tukey, *P*=0.96, Fig. 3). The migration intensity of exposed fish was steadily decreasing over time, which is in line with the elimination rate of oxazepam from fish muscle tissue (Fig. 4). The no-effect concentration occurred after approximately 60 h at when mean fish muscle concentration of oxazepam was about 7.0 μg kg^−1^ (Fig. 4).

### Diurnal trends

**Figure 5:**
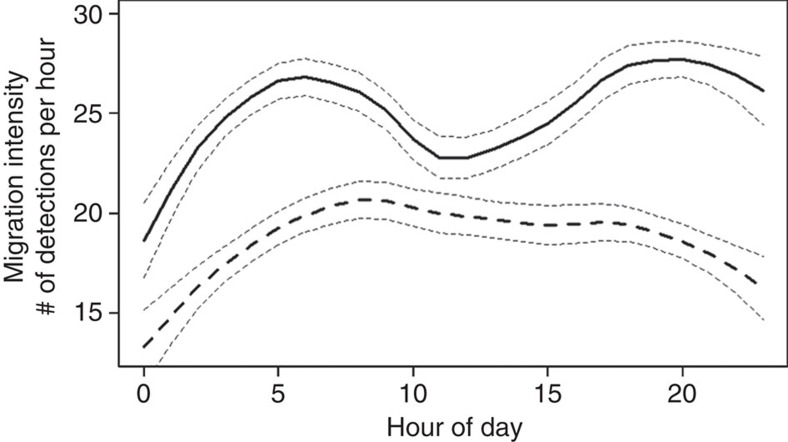
Diel migration activity in Atlantic salmon smolt. Diel migration activity for control (dashed line) and oxazepam-exposed (solid line) Atlantic salmon smolt. Migration intensity was measured as the mean number of downstream detections per hour per fish individual. The lines are embedded with 95% confidence interval bands, and are the fitted values from a Loess smoother.

There was a clear difference between exposed and control smolt in diurnal migration intensity, as revealed by LOESS smoothers fitted to diurnal migration data from the laboratory experiment (Fig. 5). However, based on model selection using Akaike information criterion, a fourth order polynomial regression model was judged the most parsimonious for both exposed and control smolt, indicating that both groups exhibited a bimodal diurnal migration rhythm, where migration occurred more intensively in the morning and the evening (Supplementary Table 1).

### Fish condition

There were no differences in mass, length, or Fulton condition factor (*K*) between control and exposed fish for neither 1- nor 2-year old smolt (one-way analysis of variance, all *P*>0.05).

## Discussion

Salmon migration is one of the most intensely researched topics in fisheries science^9^,^21^. Yet, despite the great number of studies on the physiological and environmental effects underlying migration, considerable variation in migration behaviour remains unexplained^4^,^6^. Our study clearly shows that exposure to the GABA_A_ receptor agonist oxazepam, an anxiolytic pharmaceutical, significantly increased migration intensity in salmon smolt. Although oxazepam also accentuated the diurnal migration intensity, it did not alter the diurnal migration pattern compared with the control fish or to the natural diurnal pattern seen in salmon in the adjacent Ume river system (Supplementary Fig. 1). The fact that the diurnal migration pattern in our study mimics that seen in wild salmon migrating in Ume River corroborates that we truly are measuring migration and not just activity.

Exposure to oxazepam is known to reduce anxiety in fish (reviewed in refs 17, 22), resulting in increased risk taking^23^,^24^, and the role of the GABA_A_ receptor in regulating anxiety in salmon has recently been shown using gabazine, an antagonist to the GABA_A_ receptor^25^. Hence, the intensified migration seen in our study could be explained by a reduction in anxiety that otherwise would constrain the intensity of risky activities such as migration. Downstream migration behaviour is crucial in the life cycle of salmon, and the timing and intensity of the migration is adaptive as it impacts survival and fitness of the fish^6^,^26^. An emotional adaptation^27^, where selection acts on anxiety phenotypes that generates a migration response matching the specific risk conditions prevailing in the river or estuary, is an intriguing and novel thought that complement current research stating migration as strongly governed by the cognitive ability and personality of fish^11^,^28^.

Any disruption in salmon migration behaviour can have unforeseen and severe ecological consequences^6^. Recently, the anxiolytic function of the GABAergic system has gained attention in ecotoxicology and animal conservation, as anthropogenically altered environmental conditions, such as pharmaceutical contamination and acidification, have been found to affect the GABA_A_ receptor in fish with subsequent behavioural and potentially ecological effects^17^,^29^. Anxiolytic drugs targeting the GABAergic system enter wastewater effluents at concentrations high enough to affect behaviour in fish^24^,^30^. In our study, salmon smolts were exposed to concentrations of oxazepam within the range of concentrations that have been found in effluent water^31^,^32^, and we here show effects of dilute concentrations of pharmaceutical on behaviours linked to crucial non-reproductive life cycle events in fish. The use of anxiolytic pharmaceuticals is projected to increase globally^33^, and, as a consequence, concentrations of oxazepam in aquatic environments close to urbanized areas may more than double in the coming decades^34^. With such scenarios in mind, significant ecological effects of benzodiazepines in the environment seems plausible, especially as concentration of oxazepam in fish tissue may be magnitudes higher than in the surrounding water due to bioconcentration^24^,^35^. As such, this study highlights the importance of understanding the behavioural and ecological implications of such contamination to make proper ecotoxicological risk assessments^36^.

In addition to anxiolytic pharmaceuticals, recent research has shown water pH to influence the polarizing state of the GABA_A_ receptor in fish, with behavioural alterations as a consequence^29^. In waters acidified by dissolved CO_2_, the action potential of the GABA_A_ receptor is reduced, leading to reduced anxiety in fish, often expressed as an increase in risk taking^37^,^38^. Acidification of waters is emphasized as a major global threat to salmon, based on the negative impact on physiological development and survival^39^. Recently, climate-change related acidification, due to increased CO_2_ levels, has been suggested to be especially detrimental to salmon^20^, partly because it modifies risk-taking behaviour and anxiety during the freshwater phase^25^. Our study adds to this concern by suggesting that also migration behaviour could be affected. However, it is important to be aware that although oxazepam and CO_2_ exposure lead to the same endpoint (that is, modification of GABA_A_ receptor function and altered anxiety levels), we still lack knowledge of comparable levels of effect concentrations for oxazepam and CO_2_ exposure. Further, CO_2_ exposure may also have considerably different effect dependent on environment and species. For example, fish species evolved in well-buffered systems such as the ocean may be more sensitive to variation in pH compared with fish in freshwater environments where seasonal fluctuations in pH may be significant. Nevertheless, pH effects on the GABA_A_ receptor has recently been demonstrated for freshwater fish species, indicating that alterations of the carbonate buffer system in aquatic ecosystems might have broad-scale effects on fish behaviour^40^. However, more knowledge of potential consequences of GABA_A_-receptor-induced modification in migration intensity due to changes in pH following anthropogenic-induced acidification or liming is needed.

Although the ecotoxicological relevance of our findings is evident, the fact that we found significant increases in migration following exposure to oxazepam suggests direct applications in fisheries management. Enhancing weak salmonid populations by stocking hatchery-reared smolt is a common management practice worldwide^41^, but the success of such enhancement stocking is considered to be low^42^,^43^. Inadequate migration behaviour in hatchery smolt has been identified as an important reason for the low success^44^,^45^,^46^,^47^, as low migration intensity increases the risk of predation^5^,^10^. Consequently, large efforts have been made to improve migration intensity of hatchery fish, via, for example, enriched rearing environments^48^, intense exercising programs^47^, and dietary regimes^49^, but with so far modest success. It is noteworthy that by exposing the hatchery smolt to low concentrations of oxazepam, the smolt almost doubled its migration intensity. Such treatment, although unconventional, would not drug the fish permanently as elimination rate of oxazepam is relatively high as shown in this study. Future studies should investigate how oxazepam exposure affects migration success of hatchery smolt after release into rivers, using suitable telemetry techniques to track their behaviour and survival in large natural systems^50^.

Our study adds important new mechanistic understanding of migration behaviour in salmon by suggesting the involvement of the GABA_A_ receptor for regulation of downstream migration intensity. The study showcase a novel method of using psychoactive drugs to investigate the role of anxiety for fish behaviour and ecology, and provides a new conceptual framework for understanding salmon migration by suggesting cognitive and emotional dimensions that complement the current view of migration as a purely environmental and physiological response. Hence, our findings have implications for aquatic ecotoxicology and conservation, as well as for applied fisheries management.

## Methods

### Experiment

All experimental protocols and animal handling in this study were approved by the Swedish Animal Ethics Board (Dnr: A-11-13). The laboratory study was conducted between 6th of May and 20th of June 2013 and the field study between 27th May and 1st of June 2015, at Norrfors compensatory hatchery (Vattenfall AB) (63°52′ N; 20°01′ E), located at the Ume River about 10 km from the city of Umeå, Sweden. The hatchery annually produces 80,000 Atlantic salmon smolts, which are released into the Ume River as compensation for the loss of wild smolt production due to hydroelectric damming of the river. The smolts are reared in large round pools (diameter 8 m, depth 1 m), supplied with continuously running water diverted from the nearby river, and are fed according to standard commercial protocols. The light conditions in the hatchery are set to follow the natural light regime for the region. The majority of the fish smoltify at the age of 1 or 2 years. During late spring, the young salmon (that is, parr) stops holding a position against the current and start to actively swim downstream. This change in behaviour signals the completion of the smolting^51^ and is used by the hatchery staff as a cue to initiate the release of salmon into the river^52^.

### Laboratory study

In April, before the onset of the downstream migration, 80 2-year old and 120 1-year old salmon were randomly selected from the hatchery stock and transferred to 400 l holding tanks supplied with continuously running water. One tank per age group was used. In conjunction with the transfer, the parr were anaesthetized with MS222 and measured for length (mean±1 s.d.: 1-year old=114±8.5 mm, 2-year old=145±8.3 mm) and weight (mean±1s.d.: 1-year old=14.5±3.6 g, 2-year old=26.9±7.3 g), after which each fish was tagged with a 12-mm passive intergrated transponder (PIT) (Biomark Inc, BIO12.B.03/TX708HQ) into the abdominal cavity using a syringe. PIT tags are routinely used to track and monitor juvenile salmonids, with very little or no impact on the fish^53^.

### Exposure (laboratory)

The experiment was run in a set of six trials, each trial including 96 h of exposure to oxazepam, and 70 h of migration test. Forty fish (20 that had been exposed to oxazepam and 20 unexposed controls) were tested in each of the first four trials, while 20 control fish were tested in the fifth trial, and 20 exposed in the sixth trial. Every third day, starting 1st of June, a new trial was initiated. The first two trials consisted of two-year old fish, and the last four trials of 1-year old fish. Exposure started by transferring fish from the holding tanks to 1 × 1-m cattle tanks filled with 280 l of standing water diverted from the river, keeping 20 fish per tank. Then, oxazepam dissolved in water was poured into one of the cattle tanks resulting in an oxazepam concentration of 1.9 μg l^−1^. Each cattle tank had an oxygen pump to compensate for the oxygen consumption by the fish. Water temperature was kept constant at 10 °C during the exposure period. Initial studies on the uptake and elimination of oxazepam in salmon smolt showed that salmon reach steady state with the surrounding water concentrations after 4 days (Supplementary Fig. S2), and the fish in this study were hence exposed for 96 h to ensure steady state. The same initial uptake study also concluded that salmon completely eliminated oxazepam from blood and tissue after 150 h if kept in clean water (Supplementary Fig. S2). After 60–70 h in clean water, tissue-concentrations of oxazepam are below 7.5 μg kg^−1^, which is the lowest concentration known to generate behavioural modifications in fish in earlier studies^30^. Based on this, each trial in the migration study (see below) was limited to 70 h. Water samples were taken continuously throughout the study to monitor oxazepam concentrations.

### Laboratory based migration assay

**Figure 6:**
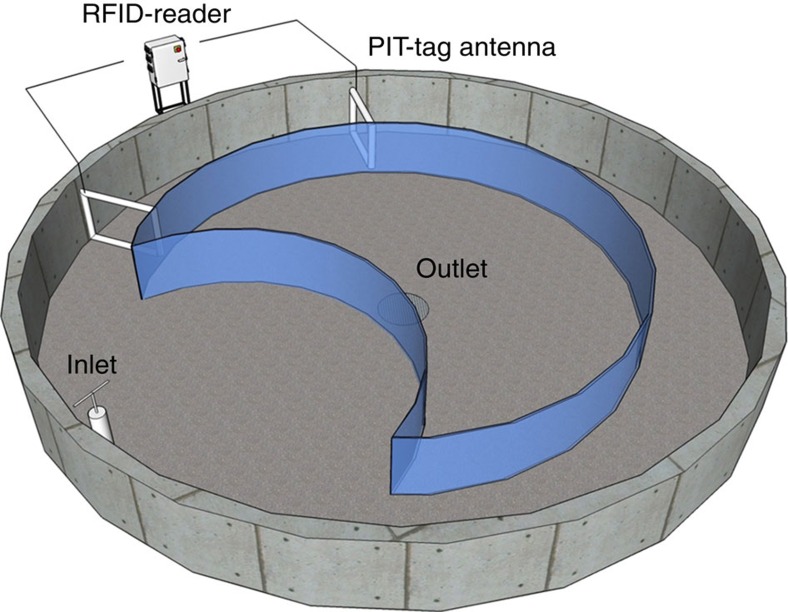
Schematic drawing of the migration mesocosm used to monitor downstream migration. The centre of the rearing tank (diameter 8 m) is enclosed, restricting fish from entering the area. At the narrowest passages between the enclosed area and the tank wall, two PIT-antennas were installed, connected to a reader station. A ‘dent' in the enclosed area created a back eddy, serving as a refuge for the fish from the water current. Schematic drawing by G Hellström.

After exposure, fish were moved into a migration study pool (diameter 8 m, depth 1 m, see Fig. 6). Two pools, one per treatment, were used and treatments were shifted between pools for every trial to avoid pool-generated effects. As with the rearing pools, the study pools were continuously supplied with water from the Ume River, keeping water temperatures identical to the natural regime of the season. The continuous flowthrough of water created a unidirectional water current in the pools, with water circulating alongside the walls. The centre of each pool was enclosed, restricting fish from entering the central area (Fig. 6). A ‘dent' in the enclosed area created a back eddy, which served as a refuge for the fish from the current. At the narrow passage between the enclosed area and the wall of the pool, two PIT-tag antennae, 3 m apart, were installed and connected to a reader (Biomark/Allflex RM310). Fish passing through these antennae were registered on the reader. Each registration included the tag ID (unique for each fish) and the time and date of detection. The reader collected data non-stop during the study period and a higher number of detections indicate higher migration intensity. A similar circular migration-pool setup has successfully been used in earlier studies of migration behaviour in Atlantic salmon with regards to swimming intensity and speed, and motivation to migrate^52^,^54^,^55^,^56^,^57^. After the experiment, all fish were released into the Ume River.

### Analysis

Salmon were allowed to acclimatize for 30 min in the pools; only data recorded after the acclimation period were used in subsequent analysis. The difference in downstream migration intensity between exposed and control smolt was analysed using a general LME model, treating number of detections per fish as response variable and treatment (exposed/control) as a two-level factor. Trial nested in age was included as a random effect structure, to account for dependence between observations within age and trial. Gaussian error was used, as both the response variable and the model residuals were found to be normally distributed by visual inspection. Statistical inference on the treatment effect was done using Wald test. To investigate the dependency of time on the effect of Treatment on migration intensity, difference between exposed and control fish was tested for the first 10 h (0–10) and the last 10 h (60–70) using a similar model structure as above, but also including the interaction term ‘treatment × time' (10 first/10 last hours). To investigate the continuous decline of migration intensity over time and in relation to the elimination rate of oxazepam from muscle tissue, a LOESS smoother^58^ was fitted for each treatment and graphically compared in a plot, having time (0–70 h) on the *x* axis and number of detections per hour (migration intensity) on the *y* axis. A LOESS smoother was also used to investigate difference between treatments in diurnal migration intensity using hour of day on the *x* axis and number of detections (migration intensity) on the *y* axis. To more formally determine the form of the relationship between migration intensity and hour of day, multi-order polynomial regression models were fitted to the data, and the most parsimonious of these models was determined using Akaike information criterion-based model-selection.

Potential differences between treatments in mass, length and Fultons condition index^59^ were assessed using one-way analysis of variance. All analyses were performed in the statistical program R^60^, using the package nlme^61^.

### Field-based migration assay

The field study was carried out in a small stream (0.5 m^3^ s^−1^) that enters into Ume river close to the hatchery (63°52′ N; 20°01′ E, Supplementary Fig. S3). The procedure to select and tag smolt was identical to the laboratory study described above. For exposure 200 1-year old smolts (length mean±1 s.d.:=114±15 mm, weight mean±1 s.d.:=14.8±4.3 g) were evenly distributed into four 1 × 1-m tanks, resulting in 50 smolts per tank. Fish in two of the tanks were exposed to 1.9 μg l^−1^ oxazepam, while the other two were kept as controls containing unexposed fish in river water. After a seven-day exposure period, the smolts were released into a small stream pool 300 m upstream from where the stream enters the river. A migration barrier immediately upstream the pool prevented the fish from moving further upstream. Fifty smolts form each treatment were released at two occasions, at 09:30 and 11:30 on 27 May 2015. A PIT-tag antenna connected to a reader (Biomark HPR Plus) was installed 200 m downstream of the release pool one week before releasing the smolts (Supplementary Fig. S3). The antenna covered the entire width of the stream, and, hence, all migrating smolts had to pass through the antenna on their way downstream. The antenna was removed 3 days after the smolts were released, and during those three days 39% of the control and 71% of the exposed smolts had migrated pass the antenna. Smolts that had not been detected during that time were not considered in subsequent analysis.

Time from release to first detection on the antenna was treated as a normal distributed response variable in a general linear mixed effect model, having Treatment as a two-level fixed effect (exposed, control) and Release occasion as a two-level random effect (first and second release occasion).

### Data Availability

The data supporting this study are available on request from the corresponding author

## Additional information

**How to cite this article**: Hellström, G. *et al*. GABAergic anxiolytic drug in water increases migration behaviour in salmon. *Nat. Commun.* **7**, 13460 doi: 10.1038/ncomms13460 (2016).

**Publisher's note**: Springer Nature remains neutral with regard to jurisdictional claims in published maps and institutional affiliations.

## Supplementary Material

Supplementary Information Supplementary Figures 1-3, Supplementary Table 1
