# Supplementary material for: GABAergic anxiolytic drug in water increases migration behaviour in salmon
Source: Nat Commun. 2016 Dec 6;7:13460. doi: 10.1038/ncomms13460 (PMC5155400; doi:10.1038/ncomms13460)
Supplement: Supplementary Information — Supplementary Figures 1-3, Supplementary Table 1 [file ncomms13460-s1.pdf]

Supplementary Information

Supplementary Figures

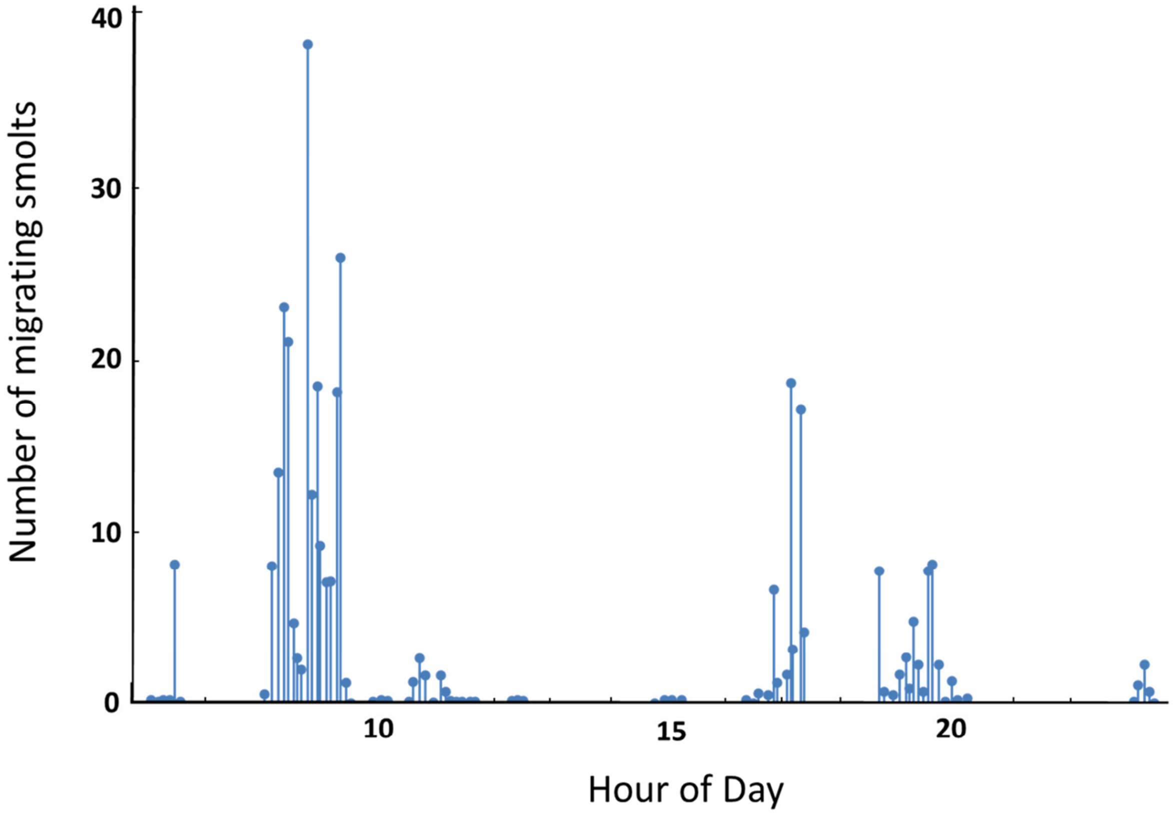

Figure S1. Mean number of downstream migrating wild Atlantic salmon smolt per 5 minutes in the main river at the study site: Mean number of downstream migrating wild Atlantic salmon smolt per 5 minutes, passing through the intake of a fish ladder at Stornorrfors power station, Ume River (63°52´ N; 20°01´ E), from the 26th to the 29th of June, 2015. The smolt was manually counted from continuous video recordings filmed through an observation window at the intake of the fish ladder.

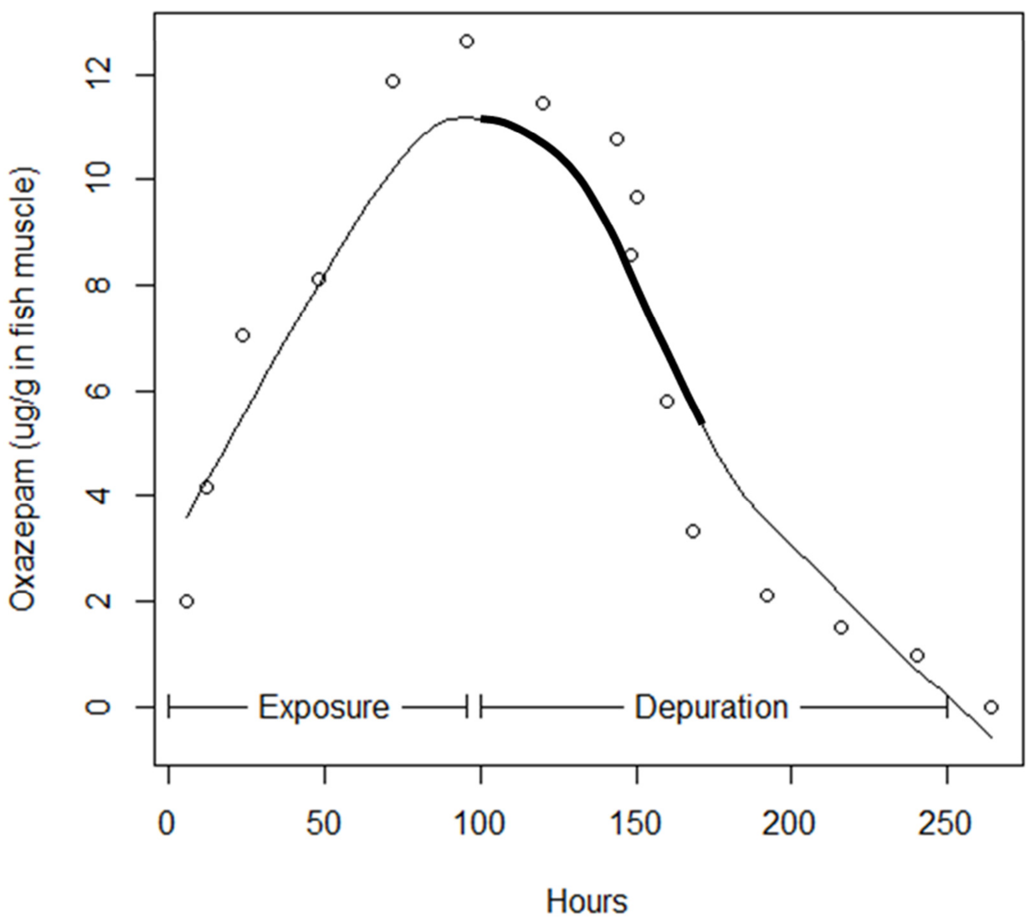

Figure S2: Muscle tissue concentrations of oxazepam, during the uptake and depuration phases, in Atlantic salmon smolt. Concentrations of oxazepam in Atlantic salmon smolt muscle tissue following exposure to water with 1.9 µg oxazepam L^{-1}, showing uptake during the first 96 hours and the following depuration phase. Rate of excretion over time (i.e. depuration) was quantified after placing the salmon smolt in clean water. Fish muscle-tissue samples were analysed according to methods described in detail in Brodin et al. (2013). In short, fish muscle samples (0.1 g) were extracted sequentially using a mixture of methanol and water, formic acid, acetonitrile and acetonitrile. Extracts were analysed using a triple stage quadrupole MS/MS TSQ Quantum Ultra EMR (Thermo Fisher Scientific, San Jose, CA, USA) coupled with an Accela LC pump (Thermo Fisher Scientific, San Jose, CA, USA) and a PAL HTC autosampler (CTC Analytics AG, Zwingen, Switzerland). The fat part of the depuration line represent the 70 hour study period.

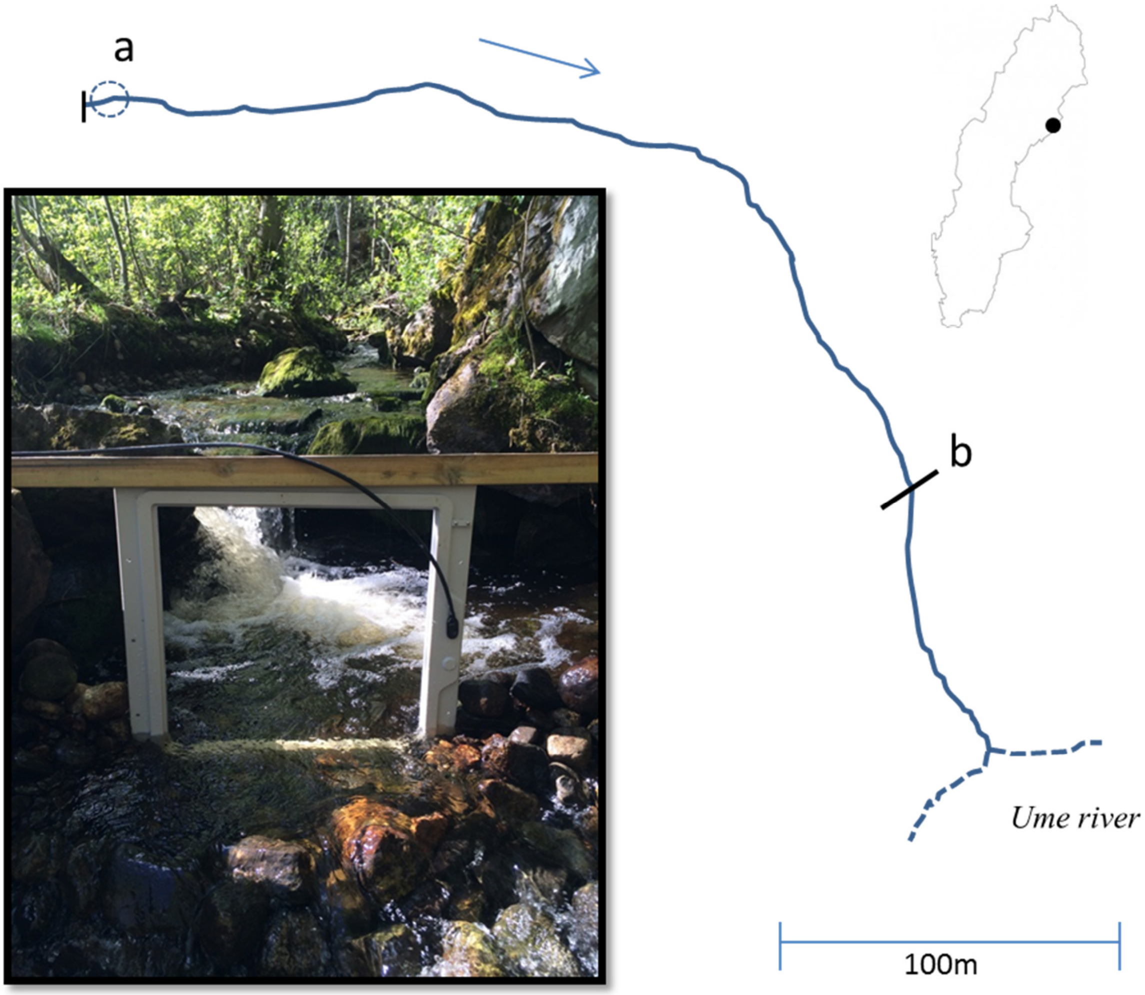

Figure S3. Schematic illustration of the river tributary used in the field study: Field study river tributary with a) is the pool in which the fish were released and b) is the location of the PIT-tag antenna used to detect migrating smolt. The insets show location of site in Sweden (upper right) and PIT-tag antenna at the site (lower left). Photo by G Hellström.

Exposed

	ΔAIC
$MigInt = \alpha + \beta_1 * Hour + \beta 2 \quad * Hour^2 + \beta_3 * Hour^3 + \beta_4 * Hour^4$	0
$MigInt = \alpha + \beta_1 * Hour + \beta_2 * Hour^2 + \beta_3 * Hour^3$	136
$MigInt = \alpha + \beta_1 * Hour + \beta_2 * Hour^2$	145
$MigInt = \alpha + \beta_1 * Hour$	145

Control

	ΔAIC
$MigInt = \alpha + \beta_1 * Hour + \beta_2 * Hour^2 + \beta_3 * Hour^3 + \beta_4 * Hour^4$	0
$MigInt = \alpha + \beta_1 * Hour + \beta_2 * Hour^2 + \beta_3 * Hour^3$	7
$MigInt = \alpha + \beta_1 * Hour + \beta_2 * Hour^2$	7
$MigInt = \alpha + \beta_1 * Hour$	44

Table S1: Summary of models describing the relationship between Migration Intensity (*MigInt*) and Hour of day (Hour) for oxazepam-exposed and control Atlantic salmon smolt. Models are ranked based on the change (Δ) in Akaike Information Criterion (AIC) compared to in the most parsimonious model.
